# Supplementary material for: Color-Specific Recovery to Extreme High-Light Stress in Plants
Source: Life (Basel). 2021 Aug 10;11(8):812. doi: 10.3390/life11080812 (PMC8398727; doi:10.3390/life11080812)
Supplement: Supplementary file 1 [file life-11-00812-s001.zip › life-1252701-supplementary.pdf]

*Supplementary data of:*

# Color-Specific Recovery to Extreme High-Light Stress in Plants

**Débora Parrine [1,†], Todd M. Greco [2], Bilal Muhammad [3], Bo-Sen Wu [1], Xin Zhao [3] and Mark Lefsrud [1,*]**

[1] Department of Bioresource Engineering, Macdonald Campus, McGill University, 21,111 Lakeshore
Boulevard, Sainte-Anne-de-Bellevue - QC, H9X 3V9, Canada;
debora.parrine@imbim.uu.se (D.P.);
bo-sen.wu@mail.mcgill.ca (B.-S.W.)

[2] Department of Molecular Biology, Princeton University, Princeton, NJ 08544, USA;
tgreco@princeton.edu

[3] Department of Animal Science, Macdonald Campus, McGill University, 21,111 Lakeshore Boulevard, Sainte-Anne-de-Bellevue - QC, H9X 3V9, Canada;
muhammad.bilal@mail.mcgill.ca (B.M.);
xin.zhao@mcgill.ca (X.Z.)

* Correspondence: mark.lefsrud@mcgill.ca

† Current address: Institutionen för Medicinsk Biokemi och Mikrobiologi, Biomedicinskt Centrum, Uppsala Universitet, 752 37 Uppsala län, Uppsala, Sweden.

## Non-photochemical quenching measurements

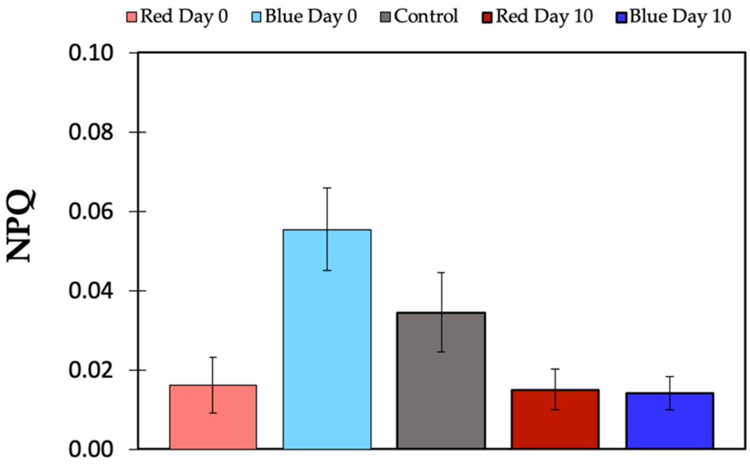


**Figure S1.** Measurements of non-photochemical quenching (NPQ) obtained from the Burned zone of a tomato leaf after a Red or Blue light treatment (RLT and BLT, respectively). Changes in parameters from tomato plants (*Solanum lycopersicum*) stressed with deep-red (655 nm) or blue (470 nm) LEDs at ~5,000 W $m^{-2}$ intensity and control (no high light treatment). Data points from "Day 0" were obtained immediately after the high light treatment, and "Day 10" data points were collected 10 days after the treatment. Induction of non-photochemical quenching (NPQ), which is calculated by NPQ= $F_M/F_{M'} - 1$. Vertical bars indicate the standard error (SE) of the means (n=3). Means are significantly different at p<0.01, or p<0.05 when indicated by (*), according to the Tukey's multiple comparison tests.

*NPQ measurement methodology*

Chlorophyll (Chl) fluorescence measurements were performed using a leaf chamber fluorometer (LI-6400-40, LI-COR Inc., Lincoln, NE, USA). Measurements were done in triplicates, for obtaining the photochemical

efficiency of PSII (Fv/Fm) of dark-adapted leaves. The measurements were performed following previously established guidelines [18]. Briefly, a modulated red radiation of approximately 2 μmol $m^{-2}$ $s^{-1}$ was used to excite fluorescence by using a frequency and a pulse width of 20 kHz and 3 μs, respectively. About 8,000 μmol $m^{-2}$ $s^{-1}$ saturating radiation pulse of 0.8 s was utilized. The open PSII center (F0) minimum Chl fluorescence and the closed PSII center maximal Chl fluorescence values were obtained after a 20 min dark-adaptation period. After, leaves were irradiated continuously, and the steady-state fluorescence (Fs) was determined. A new 8,000 μmol $m^{-2}$ $s^{-1}$ saturating pulse was emitted for obtaining the maximal fluorescence of light-adapted state (Fm′). Then, the actinic PPFD was turned off and a far-red (740 nm) light was used to measure the minimum fluorescence of light-adapted state (F0′). The obtained values were used to calculate the following: i) Fv/Fm = (Fm − F0)/Fm, the maximum dark-adapted PSII photochemical efficiency; ii) ΦPSII = (Fm′ − Fs)/Fm′, the effective light-adapted photochemical efficiency; iii) qP = (Fm′ − Fs)/(Fm′ − F0′), the photochemical quenching; iv) NPQ = (Fm − Fm′)/Fm′, the nonphotochemical quenching; and v) ETR = ΦPSII × 0.5 × 0.84 × PPFD, the PSII electron transport rate.
